# Recovery of device‐measured physical activity after major abdominal surgery in older adults

**DOI:** 10.1111/anae.70310

**Published:** 2026-07-21

**Authors:** Daniel Rubin, Kyle Tingling, Megan Huisingh‐Scheetz, Nancy W. Glynn

**Affiliations:** ^1^ University of Chicago Chicago IL USA; ^2^ University of Pittsburgh Pittsburgh PA USA

For many older adults undergoing surgery, return to baseline physical function and independence are primary recovery goals [1]. Major abdominal surgery is a physiological stressor, and older adults often have less muscle mass and lower baseline physical activity, which places them at increased risk of delayed recovery [2]. Despite the importance of functional recovery to patients, the expected timeline for return to baseline of daily physical activity after surgery remains poorly characterised [3]. Patients are told that recovery may take up to 3 months, although device‐measured data supporting this timeframe are limited. We, therefore, sought to characterise recovery trajectories of daily physical activity following abdominal surgery using wearable accelerometers.

Older adults (aged ≥ 60 y) were enrolled prospectively from the peri‐operative clinic at the University of Chicago Medical Centre, Chicago, IL, USA. Patients were eligible if they were scheduled for abdominal surgery > 7 days after their visit and were able to ambulate with or without an assistive device. Non–English‐speaking patients were not studied. The study was approved by the University of Chicago Institutional Review Board, with all patients providing written informed consent.

Study procedures included: collection of demographic information; comorbidities; Duke Activity Status Index; the Mini‐Cog; Fried Frailty Phenotype scoring; timed five sit‐to‐stands; and accelerometer‐based activity monitoring [4, 5, 6]. Surgical complications were recorded from the time of surgery to 30 postoperative days. Physical activity was measured using a wrist‐worn ActiGraph wGT3X‐BT accelerometer (Ametris, Pensacola, FL, USA). Patients were instructed to wear the device continuously on their non‐dominant hand for 7 days at four time points: pre‐operatively (baseline); at hospital discharge; and at 1 month and 3 months postoperatively.

Accelerometer data were processed using GGIR in R Studio (Posit Software, PBC, Boston, MA, USA). Moderate physical activity was categorised by raw accelerations using cutpoints according to Migueles et al. [7] and step counts were derived using the modified Verisense algorithm [8]. A valid day required at least 16 h of wear time and at least 3 valid days were required for inclusion. Mixed‐effects regression models included fixed effects for pre‐operative frailty and surgical complications within 30 postoperative days. Patient‐level random effects were used to evaluate changes in physical activity relative to baseline. Recovery to baseline was defined as activity levels that were not statistically different from baseline.

A total of 70 patients were enrolled, of whom six withdrew before surgery and nine had no accelerometer wear time; thus, 55 patients were included in the final analysis. The mean (SD) age was 69.7 (10.6) y, 21 patients were prefrail or frail with a mean (SD) gait speed of 0.98 (0.2) m.s^‐1^ and 14 developed a complication within 30 postoperative days (Table 1). At baseline, the median (IQR [range]) daily step count was 4737 (2837–6415 [382–17,154]) steps.day^‐1^. Following surgery, step counts at discharge, 1 month and 3 months were 1425 (746–3027 [92–9407]), 2762 (1788–5279 [90–16,161]) and 4356 (2375–6159 [929–13,039]) steps.day^‐1^, respectively (Fig. 1a). Only daily step count at 3 months was not significantly different from baseline (p = 0.343) (online Supporting Information Table S1).

**Table 1:** Patient characteristics. Values are mean (SD) or number.

	Patients
	n = 55
Age; y	69.7 (5.3)
Sex; female	29
Ethnicity	
White	39
Black	12
Asian	2
Unspecified	1
BMI; kg.m^‐2^	29.0 (12.4)
Diabetes mellitus	8
Coronary artery disease	8
Peripheral vascular disease	2
Cerebral vascular disease	1
Hypertension	32
Heart failure	4
Chronic kidney disease	6
Requires assistive walking device	3
Fried Frailty Phenotype	
Not frail	34
Pre‐frail	17
Frail	4
Usual gait speed; m.s^‐1^	0.98 (0.4)
Time to perform 5‐sit to stand; s	13.3 (6.8)
Mini‐Cog score (≥ 3)	53
Duke Activity Index score	45 (28)
30‐day surgical complications	14

**Figure 1:**
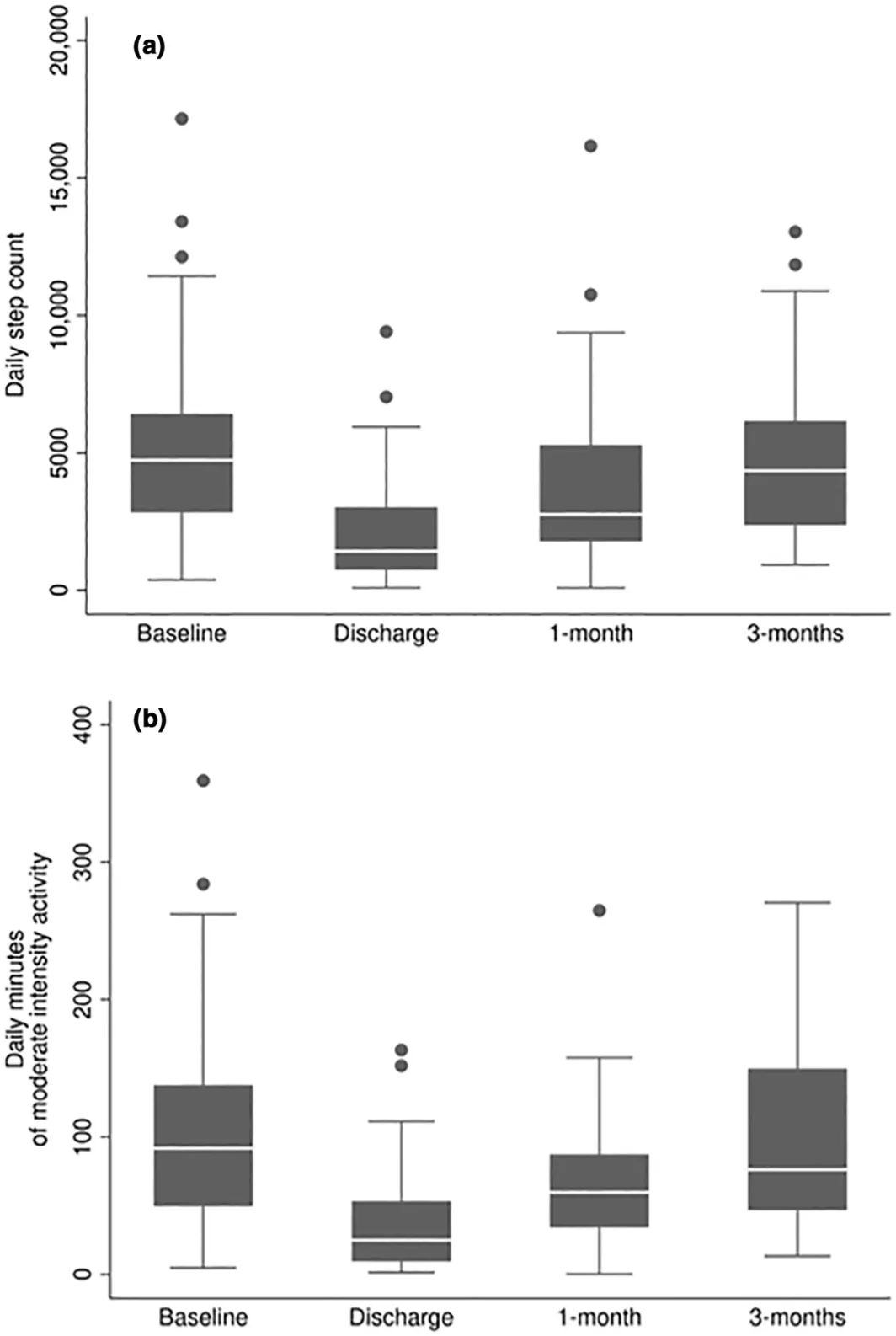
Postoperative changes in device‐measured physical activity relative to baseline across all study time points. Box‐and‐whisker plots show the median (white line), interquartile range (box), 1.5 × interquartile range (whiskers) and outliers (individual points). (a) Daily step count; (b) moderate‐intensity physical activity.

Time spent in moderate‐intensity activity showed a similar trajectory. Median (IQR [range]) moderate activity at baseline was 92 (50–138 [5–359]) min.day^‐1^. Postoperative values at discharge, 1 month and 3 months were 25 (10–53 [1–163]), 60 (34–87 [0–265]) and 76 (47–150 [13–270]) min.day^‐1^, respectively (Fig. 1b). Only moderate activity level at 3 months was not significantly different from baseline, p = 0.826 (online Supporting Information Table S2). Baseline frailty status and 30‐day complications were not associated with recovery of daily step count or time in moderate activity.

In this prospective cohort study, older adults who were largely non‐frail and pre‐frail and undergoing abdominal surgery experienced declines in physical activity immediately after surgery. This then returned to baseline activity levels by 3 months. The volume of activity (daily step count) and intensity of activity (time spent in moderate activity) followed similar recovery trajectories and were not modified by baseline frailty category or complications within 30 postoperative days. These findings provide device‐measured data to inform expectations for functional recovery after surgery. Clinicians should consider discussing expected activity trajectories when counselling patients about postoperative recovery.

Our study has limitations. The cohort was at a moderate level of functioning, with only 7% meeting frailty criteria, limiting assessment of differences in recovery by frailty status. Nonetheless, a mean baseline gait speed < 1 m.s^‐1^ suggests that the cohort remained at risk. The single‐centre design and modest sample size also may limit generalisability. These findings provide novel insight into the timeline of recovery of daily physical activity following abdominal surgery and suggest that many older adults may return to baseline activity levels between 1 and 3 months postoperatively.

## Supporting information


**Table S1.** Mixed‐effects regression to evaluate recovery of daily step count.
**Table S2.** Mixed‐effects regression to evaluate recovery of daily minutes of moderative activity.
