# Supplementary material for: Recovery of device‐measured physical activity after major abdominal surgery in older adults
Source: Anaesthesia. 2026 Jul 21;81(10):1429–31. doi: 10.1111/anae.70310 (PMC13551170; doi:10.1111/anae.70310)
Supplement: Supplementary file 1 [file ANAE-81-1429-s001.docx]

**Table S1:** Mixed-effects regression to evaluate recovery of daily step count across discharge, 1-month and 3-months post recovery from major abdominal surgery.

| **Variable** | **Change in step count steps/day** | **Std. Err.** | | **z** | | **P>\|z\|** | **[95% Conf.** | **Interval]** |
| --- | --- | --- | --- | --- | --- | --- | --- | --- |
| Baseline (REF) | - |  | |  | |  |  |  |
| Discharge | -2875.83 | 332.48 | | -8.65 | | <0.001 | -3527.47 | -22224.19 |
| Month 1 | -1179.43 | 352.96 | | -3.34 | | <0.001 | -1871.25 | -487.61 |
| Month 3 | -344.20 | 363.35 | | -0.95 | | 0.34 | -1056.35 | 367.96 |
| 30-day Complications | -1116.57 | 859.11 | | -1.30 | | 0.19 | -2800.40 | 567.27 |
| Non-frail (REF) | - |  | |  | |  |  |  |
| Prefrail | -1378.19 | 824.35 | | -1.67 | | 0.10 | -2993.88 | 237.45 |
| Frail | -1568.57 | 1454.26 | | -1.08 | | 0.28 | -4418.87 | 1281.73 |
| Random-effects Parameters | | | Estimate | | Std. Err. | | [95% Conf. | Interval] |
| individual: Unstructured var(time)  var(_cons) | | | 81541 6634174 | | 130558.2 1565067 | | 3535.79 4178111 | 1880466  105000000 |
| var(Residual) | | | 2436547 | | 376373 | | 1800072 | 3298069 |

Frailty was measured using Fried frailty phenotype. 30-day complications were measured according to the National Surgery Quality Improvement Project definitions.

**Table S2:** Mixed-effects regression to evaluate recovery of daily minutes of moderate activity intensity across discharge, 1-month and 3-months post recovery from major abdominal surgery.

| **Variable** | **Change in minutes of moderate activity/day** | **Std. Err.** | | **z** | | **P>\|z\|** | **[95% Conf.** | **Interval]** |
| --- | --- | --- | --- | --- | --- | --- | --- | --- |
| Baseline (REF) | - |  | |  | |  |  |  |
| Discharge | -62.94 | 6.64 | | -9.47 | | <0.001 | -75.97 | -49.91 |
| Month 1 | -27.31 | 7.36 | | -3.71 | | <0.001 | -41.73 | -12.89 |
| Month 3 | -1.72 | 7.83 | | -0.22 | | 0.83 | -17.08 | 13.63 |
| 30-day Complications | -22.60 | 16.37 | | -1.38 | | 0.17 | -54.69 | 9.49 |
| Non-frail (REF) | - |  | |  | |  |  |  |
| Prefrail | -24.69 | 15.97 | | -1.55 | | 0.12 | -55.99 | 6.62 |
| Frail | -31.12 | 28.53 | | -1.09 | | 0.28 | -87.04 | 24.79 |
| Random-effects Parameters | | | Estimate | | Std. Err. | | [95% Conf. | Interval] |
| individual: Unstructured var(time)  var(_cons) | | | 77.98 2454.28 | | 60.02 590.79 | | 17.25 1531.17 | 352.46 3933.92 |
| var(Residual) | | | 1005.16 | | 152.26 | | 746.95 | 1352.62 |

Frailty was measured using Fried frailty phenotype. 30-day complications were measured according to the National Surgery Quality Improvement Project definitions.
